# Building Work Engagement in Organizations: A Longitudinal Study Combining Social Exchange and Social Identity Theories

**DOI:** 10.3390/bs13020083

**Published:** 2023-01-19

**Authors:** Silvia Simbula, Simona Margheritti, Lorenzo Avanzi

**Affiliations:** 1 Department of Psychology, University of Milano-Bicocca, 20126 Milano, Italy; 2 Department of Psychology and Cognitive Sciences, University of Trento, 38068 Rovereto, Italy

**Keywords:** organizational identification, social exchange, social identity, social support, work engagement

## Abstract

Starting from the insights of social identity theory and social exchange theory, the present study aimed to understand how social support and organizational identification relate to work engagement. Moreover, it sought to verify if social support and organizational identification interact with each other to explain work engagement three months later. A longitudinal study was conducted on a sample of 150 employees, in which organizational identification, social support, and work engagement were measured through a questionnaire. The results show that when employees can count on their supervisors’ and colleagues’ support, they will be more engaged in their work. In addition, when an employee strongly identifies with their organization, the employee’s evaluation of the social support received from colleagues and supervisors becomes less critical in determining their work engagement. These results confirm our hypotheses and extend the findings of previous research on withdrawal behaviors. From a practical point of view, it seems important for organizations to invest in increasing identification, as well as in building a high-quality social exchange relationship, especially when levels of organizational identification are low or decreasing.

## 1. Introduction

Occupational psychology has long been occupied with studying and defining work-related mental illnesses, such as burnout, turnover, absenteeism, and work-related stress, rather than mental wellness [1]. A significant change in the area of occupational health psychology has occurred thanks to the advent of positive psychology [2], which has shifted the focus from pathology to analyzing what is good and positive in life (e.g., in work contexts).

The need to view workplaces as spaces in which people can realize themselves and live peacefully has led researchers to study work engagement, which is defined as a positive, fulfilling, work-related psychological state that stems from the combination of three inter-related dimensions, namely, vigor, dedication, and absorption [3]. Several studies included in Mazzetti et al.’s [4] meta-analysis show that when people are engaged, they can be energetic and effective in their work and feel ready to wholly deal with the demands of their job.

Starting from studying the factors that can determine positive outcomes (i.e., work engagement), this study deepens the themes of social support and organizational identification by using insights from both social exchange theory and social identity theory. Specifically, the purpose of this contribution is twofold: firstly, to unravel the relation between social support and organizational identification, on the one hand, and work engagement, on the other hand; secondly, to provide evidence on how social support and organizational identification interact with each other to explain work engagement over time. Despite the fact the importance of social support and organizational identification for several positive organizational outcomes has already been demonstrated, the research in this field has not yet been significantly deepened, and to the best of our knowledge, most of the investigations are cross-sectional. This point is crucial because robust relations found in cross-sectional evidence could disappear when using a longitudinal design [5]. Moreover, to the best of our knowledge, no study has longitudinally examined their interaction to explain work engagement.

## 2. Literature Review

### 2.1. The Relationship between Social Support and Work Engagement

Having engaged workers represents a relevant factor for organizations because motivated and involved employees can bring advantages in terms of productivity [6,7,8], a relaxed and collaborative climate through the implementation of organizational citizenship behaviors [9,10], and less intention to quit [8].

Moreover, engagement is a significant feature because it represents a positive predisposition to work, thanks to individuals becoming promoters of their own well-being within the organization. Because of the innumerable positive impacts that this state can determine, significant research has taken into consideration several possible antecedents and all the factors that can determine its genesis. For example, several studies placed it within the theoretical framework of the Job Demands-Resources (JD-R) model [6,11], specifically within its motivational process. Following this model, job demands and personal resources allow the genesis of work engagement. On one hand, resources boost employees’ growth, learning, and improvement (intrinsic motivation); on the other hand, they are instrumental in completing job tasks (extrinsic motivation). Moreover, resources should play an important role even in the depletion process postulated by the JD-R model, acting as a buffer in the relation between job demands and negative outcomes (e.g., burnout). Several studies, even longitudinal [12,13,14], have analyzed the relationship between different resources and work engagement. One of the most investigated resources at the level of interpersonal and social relations is social support, which is characterized by the work context’s social climate involving one’s relationships with supervisors and colleagues [11,15,16]. Social support encompasses both socio-emotional and instrumental sustenance: whereas the first refers to the degree of social and emotional integration between colleagues and supervisors, the latter refers to the collaboration between co-workers and supervisors to complete work tasks [17]. A variety of beneficial effects on employees and organizations can be attained through social support. Among them, previous evidence has supported the positive association between social support and work engagement in different work contexts: for example, among Dutch telecoms managers [13], Italian teachers [18], Swedish municipal social service employees [19], Portuguese professors and support staff in public higher education [20], and Asian dispatchers [21]. Furthermore, in the study of Lockwood [22], managers’ support was the most crucial factor for workers to commit to their jobs and their propensity to keep working in the organization. Finally, several recent meta-analyses confirmed the positive association of both colleague and supervisor support with work engagement [4,14,16]. This evidence can be explained thanks to the notion of social reciprocity. When employees perceive themselves to be treated fairly and adequately (in terms of remuneration, learning opportunities, and emotional support), they should reciprocate this by putting effort into their duties and tasks [23]. Subsequently, workplace interactions are planned as mutually dependent [24] and are developed into mutual obligations and explicit rules of exchange [25]. The concept of social reciprocity fits within the theoretical framework of social exchange theory [24,25,26], which claims that employees are motivated to perform better and to carry out positive behaviors at work (for example, being more engaged) when they feel they have received sufficient treatment in a cost–benefit ratio [27,28]. Social exchange theory has been one of the major theoretical perspectives in the field of social psychology since the early writings of Homans [26], Blau [24], and Emerson [25,29]. According to the fundamental definition of social exchange theory, individuals decide by weighing the benefits and drawbacks of a situation or course of action, consciously or unconsciously, to maximize their reward.

Homans [26] defined social behavior as an exchange of time, money, effort, approval, status, and power, among other material and immaterial goods. Each individual generates benefits and incurs expenses. People will decide to perform the acts that are most likely to result in a reward equal to what they have given to another person. This idea reflects Skinner’s behavioral psychology theories about human behavior as well as the basic principles of economics [30]. Instead of emphasizing behaviorism, Blau [24] paid particular attention to the mutual exchange of extrinsic benefits, forms of affiliation, and emerging social structure produced by this type of social contact. According to Blau [24], social exchange describes human behavior driven by the benefits others are expected to provide in return for our voluntary actions, which we frequently perform. He underlines the fact that it is more common for the nature of the duties involved in social interaction to remain unclear, at least initially, when comparing it to economic exchange. The fundamental idea behind social trade is that one person does another a favor, and although there is generally some anticipation of a future reward, its specific nature is not predetermined. Like Blau [24], Emerson [25,29] considered the fundamental task of social exchange theory to be the construction of a framework in which the key variables were social structure and structural change.

Following this line of reasoning, social exchanges and relationships can generate a positive dynamic between economic and social resources present in the workplace, leading to an increase in employees’ work engagement [31].

### 2.2. The Relationship between Organizational Identification and Work Engagement

Social identity theory [32] defines social identity as the self-image that people acquire from the categories and groups to which they regard themselves as belonging. According to the theory, people build their identity through belonging to social groups and make the characteristics of their social group their own. The first reason for identifying with a group is the improvement of one’s sense of collective self-esteem; in other words, people identify with a group to provide the basis for thinking of themselves in a positive light [33].

Starting from the social identity approach as a framework, organizational identity is considered as a specific case of social identity, whereas organizational identification represents the strengths of that identity. In particular, organizational identification is defined as the “the perception of oneness with, or belongingness to the organization” [34] (p. 22). Being a member of a specific organization partially answers the questions “Who am I?” and “Who are we?” Thus, to the degree that the employees identify with their organization, they should internalize the organizational norms and goals as their own, and they should see themselves as more similar to, and therefore interchangeable with, other organizational members. Identification, in turn, should increase the collaboration among colleagues in order to achieve the best organizational performance.

What has been interesting for occupational health psychology is that organizational identification relates to (lower) stress and strain [35,36,37]. The reason is primarily linked to the satisfaction of some basic human needs such as self-esteem, but also security and belonging, and to the expansion of self-concept derived from the inclusion of connections with other people [33]. Identification also works indirectly on relational and climate aspects, since workers who feel strongly identified with their organization will perceive their colleagues as more similar, and thus more positively, which in turn should increase the likelihood of them cooperating and being helpful to each other [38]. Some scholars have specified that organizational identification is essential for an organization to work properly. For this reason, it should be one of the most crucial tasks to develop and sustain among employees [39]. Recognizing the importance of this dimension in the workplace, researchers have explored the relation of structure identification with numerous organizational dimensions, for example, turnover intentions [40,41], ethical leadership [42], job satisfaction [43], job insecurity and performance [44], and reduction in psychological distress over time [45]. Summarizing the existing research validated by meta-analyses [46,47,48], strongly identified employees should show, as a consequence, more commitment to and involvement in their organization and demonstrate greater positive attitudes and behaviors such as job satisfaction [41,49], organizational citizenship behaviors [49], and work engagement [50]. Indeed, strongly identified employees tend to work harder and put effort into achieving the organizational goals and aims because they become their personal goals and aims [45]. Because they believe organizational success is useful for personal growth, employees with a high degree of identification experience satisfaction when engaged in their work [51], and this belief is advantageous for both the development of the employees and the organization [52].

Despite most scholars agreeing that work engagement includes an energy dimension and an identification dimension, research examining the association between organizational identification and work engagement is scarce. Indeed, few studies have investigated the potential relationship of organizational identification with work engagement as an outcome variable. For example, in a recent meta-analysis on the tourism sector, Kanjanakan et al. [53] found only five papers studying the relation between organizational identification and work engagement. Ötken and Erben [39] conducted a study on a sample of 212 employees in the private sector in Istanbul, Turkey. Their study’s findings showed a positive and significant association of organizational identification with each dimension of work engagement, such that employees who strongly identified with their organizations reported higher levels of work engagement. Specifically, employees recognize their job as meaningful and challenging when they agree with the organization’s values and goals, and they feel a sense of cohesion with their organization. All these aspects lead workers to be determined at work and feel happy, powerful, and mentally resilient [39]. Moreover, Karanika-Murray et al. [43] demonstrated that employees who feel like a member of an organization sharing values that also reflect the definition of their identity tend to be more engaged with their work. Although in their meta-analysis, Kanjanakan et al. [53] found a strong and positive relation between identification and work engagement, to our knowledge, most of the studies in this field are cross-sectional, and the research has not yet been greatly deepened [43].

### 2.3. The Interaction between Social Support and Organizational Identification

As previously mentioned, according to both social exchange theory and social identity theory, people who are supported by colleagues and supervisors and identify with their organization should have a higher level of work engagement. However, these two features may also interact with each other in determining these outcomes. For example, Ötken and Erben [39] found a stronger relationship between organizational identification and work engagement when employees received positive feedback and advantages from their supervisors. Specifically, these authors found that supervisor support was a moderator of the relationship between organizational identification and the three dimensions of work engagement.

Engaged employees are absorbed in their job; they fully involve themselves in their tasks and duties, and, for this, they are strongly satisfied with and attached to their work. In this sense, engagement could represent the mirror attitude of those who instead want to leave their organization, due to the fact they are dissatisfied or stressed. In previous empirical evidence about withdrawal behaviors, van Knippenberg, van Dick, and Tavares [50] found that, with a higher level of identification with their organizations, the employees’ assessment concerning the fairness of organizational treatment had a lower weight in defining their behaviors. Therefore, in the presence of an unsatisfactory exchange relationship with the organization, employees may be engaged in the workplace and not exhibit withdrawal behaviors [54]. In fact, the study of van Knippenberg and colleagues [50] shows that only when employees have not identified with their organization can an increase in their perceptions of organizational support reduce negative behaviors (turnover and absenteeism), whereas for the employees who strongly identify, no relationship is found between support and these behaviors. Similarly, Avanzi and colleagues [54] found that organizational identification buffers the relationship between organizational support and withdrawal behaviors (i.e., turnover intentions). In particular, these authors demonstrated that high identification made the presence of (low) social support less salient. Alongside the development of these concepts, the association between supervisor and colleague support and withdrawal behaviors was weaker for strongly identified employees [50,54].

Since identification reflects a partial overlap between employees and their organization, withdrawal from the job could be seen as a threat of one’s own self-concept, and this should disincentivize employees from quitting their organization, even under poor work conditions. Likely, strongly identified employees who face low-quality relationships in the workplace prefer to actively change the situation, avoiding extreme consequences (i.e., turnover). At the same time, we hypothesize that employees experiencing positive and supportive relationships at work (i.e., high support) should evaluate this as a positive job resource able to foster their engagement. However, even facing poor work conditions, or a low level of supportive and cooperative behaviors by colleagues, strongly identified employees should not diminish their work engagement. These employees should integrate the organizational perspective with their own self-concept, and therefore they should not go against the organizational goals and aims [50]. In particular, employees who strongly identify with their organization will engage in their organization independently of the perceived and received support. In these employees, organizational values and norms should become their own standards, and, consequently, strongly identified employees will tend to embrace organizational goals and aims; for this reason, they should be more prone to work harder and to engage themselves in their jobs. However, when employees do not identify with their organizations, the transactional aspects of their job (i.e., receiving support) should become more salient. In poorly identified employees, perceiving a supportive environment represents the most important resource to push them towards engagement. Thus, following the line of reasoning of van Knippenger et al. [50] and Avanzi et al. [54], who, however, did not consider work engagement as an outcome variable, we hypothesized that organizational identification moderates the relation between perceived social support and work engagement, where the relationship is weaker for highly identified employees. This means that when employees strongly identify with their organization, their evaluation about the social support will become less important in determining work engagement.

## 3. Research Hypotheses

Focusing on this literature, we formulated the following hypotheses:

**Hypothesis 1** **(H1).** *Social support (from colleagues and supervisors) measured at Time 1 has a positive impact on work engagement at Time 2, after controlling for work engagement at Time 1*.

**Hypothesis 2** **(H2).** *Organizational identification at Time 1 has a positive impact on work engagement at Time 2, after controlling for work engagement at Time 1*.

**Hypothesis 3** **(H3).** *The relationship between perceived social support at Time 1 and work engagement at Time 2 is moderated by organizational identification at Time 1*.

## 4. Materials and Methods

### 4.1. Participants and Procedure

This study involved workers in three different organizations in Italy. The first two organizations (N = 71 and N = 27) comprised employees conducting banking operations and back-office services, which are necessary to keep a bank functioning. In the third organization (N = 52) there were employees of a social cooperative that deals with social health and educational services aimed at minors, the elderly, and people in difficulty. In the first two organizations, all the employees were asked to fill in an online questionnaire through Qualtrics. In the last organization, a paper-and-pencil questionnaire was administered. Questionnaires were matched by using anonymous codes that the respondents created from personal information, assuring their anonymity, on one hand, and allowing the university researchers to match both questionnaires to each participant, on the other hand. Before filling in the questionnaire, all the participants were asked to read and approve an informed consent form explaining the study’s aim and the procedures for the data collection. The research team assured that the answers would be confidential and anonymous. Participants were free to decide whether to participate in the study and could leave it at any time. The study was conducted in accordance with the ethical standards set by the Declaration of Helsinki. Participants were approached twice, with a time lag of approximately three months. Although no general conclusion exists about the most appropriate time lags for panel studies, several meta-analyses have shown that effects erode as the time lag between two measurements increases [55,56].

At Time 1 (T1), a total of 701 employees received the link or the paper-and-pencil questionnaire (response rate: 48%, N = 336). At Time 2 (T2), participants were asked to complete the second questionnaire (response rate with respect to T1: 44.6%, N = 150). The panel group that took part in both waves of data collection included 150 employees (68% female), with an average job tenure of 11.91 years (SD = 10.1).

### 4.2. Measures

Organizational identification was measured with the scale developed by Mael and Ashforth [57] (Italian version: Bergami and Bagozzi, [58]). Responses were given on a 5-point scale ranging from 1 = totally disagree to 5 = totally agree. A sample item is: “I am very interested in what others think about my organization”; alpha T1 = 0.69. We used only five of the six items in Mael and Ashforth’s scale because the first item (i.e., “When someone criticizes my organization, it feels like a personal insult”) worsened the Cronbach alpha value, shifting it from 0.61 to almost 0.70, representing a commonly accepted reliability cut-off.

*Work engagement* was measured by the UWES-9 [59] (Italian version: Simbula et al., [10]). All items were scored on a 7-point frequency rating scale ranging from 0 = never to 6 = always. A sample item is: “At my job, I feel strong and vigorous”; alpha T1 = 0.94 (alpha values for each dimension at T1: vigor = 0.90, dedication = 94, and absorption = 0.83); alpha T2 = 0.95 (alpha values for each dimension at T2: vigor = 0.89, dedication = 0.95, and absorption = 0.87). We followed Schaufeli et al.’s [59] recommendation and computed an overall engagement score for the UWES, which we used in the analyses.

*Social support* was measured by the HSE Management Standards Indicator Tool [60] (Italian version Toderi et al., [61]) (e.g., supervisor support: “I am given supportive feedback on the work I do”; peer support: “If work gets difficult, colleagues will help me”). Responses were given on a 5-point scale ranging from 1 = never to 5 = always; alpha T1 = 0.87. We used eight items out of the nine support dimensions because one item (i.e., I get help and support I need from colleagues) worsened the reliability of our measure. In particular, the item-total correlation test showed a very low correlation for this item (r = 0.158), and values less than 0.2 or 0.3 represent inadequate values, meaning that this item is likely not measuring the same construct measured by the other items [62].

*Covariates.* We controlled for gender and tenure because previous studies had found effects of both variables on employees’ attitudes at work (Ng and Feldman [63]). In particular, women and employees with a longer tenure tend to show more work engagement [64]. Furthermore, we also controlled for the organization (i.e., two dummy variables in which the first organization was the point of reference when constructing the dummies). Following Becker’s [65] suggestions, we also conducted all analyses with and without controls, which yielded very similar results (see below).

### 4.3. Data Analysis

Since we had a relatively small sample, we decided to test our hypotheses by using regression analysis. Specifically, the first two hypotheses were tested with two multiple linear regressions, with T2 work engagement as a dependent variable and T1 social support or T1 organizational identification as an independent variable. In both regression analyses, we also controlled for work engagement at T1. H3 was tested by means of moderation analyses by using Hayes PROCESS model 1 [66]. PROCESS is an add-on for SPSS and SAS for statistical mediation, moderation, and conditional process analysis. If the *p*-value of the interaction between variables X (social support) and M (organizational identification) is lower than 0.05, there is statistical evidence of moderation in the relationship between X and Y. The coefficients, standard errors, and 95% confidence intervals were calculated. Bootstrapped confidence intervals were used to test the interaction (5.000 resampling). By following the recommendation of Hayes and Cai [67], we ensured the robustness of our estimates by employing the HC3 estimator [67,68]. Simple slope analysis [69] was conducted on the interaction effects to reveal the nature of significant interactions and detect relations between the predictor (i.e., social support) and the outcome (i.e., work engagement) at different levels (i.e., low, medium, high) of the moderator (i.e., organizational identification). Finally, since we were testing an interaction effect and the three different organizations were small (N = 27, 52, and 71), we used the full sample by merging the sub-samples together. In fact, when testing an interaction effect, we generally had a low power, and often it was “unclear whether the interaction is not significant because the theory was wrong, or the test of the interaction lacked sufficient power” [70]. Therefore, in addition to gender, job tenure, and work engagement at T1, organization was also inserted as a covariate.

## 5. Results

### 5.1. Descriptive Statistics

The means, standard deviations, Pearson correlations, and Cronbach alpha values were calculated for all study variables (Table 1). The correlations between variables were in the expected direction. With regard to work engagement, the test–retest correlation was r = 0.81, *p* < 0.001, indicating that it was quite stable over time. Moreover, all scales showed satisfactory internal consistency.

Contrary to what is reported in the literature, job tenure was significantly negatively related to work engagement in both periods (r = −0.25 and r = −0.20, and *p* < 0.01 and *p* < 0.05 for T1 and T2, respectively), meaning that, in our samples, the length of organizational tenure decreased employees’ work engagement. Women reported more work engagement (r = 0.22, *p* < 0.01), but only at T1, whereas the correlation between gender and work engagement at T2 was not significant (r = 0.08, *p* > 0.05). As expected, the correlations between social support and work engagement were positive and significant at both times (r = 0.50 and r = 0.51, *p* < 0.01 for T1 and T2, respectively). Furthermore, identification positively and significantly correlated with work engagement at both T1 and T2 (r = 0.47 and r = 0.38, *p* < 0.01 for T1 and T2, respectively).

### 5.2. Hypothesis Testing

As predicted in H1, after controlling for T1 work engagement, social support at T1 was positively related to work engagement at T2 (B = 0.29, *p* = 0.02). Therefore, H1 was confirmed. On the contrary, the relationship between T1 organizational identification and T2 work engagement was not significant (B = 0.01, *p* = 0.92) after controlling for work engagement at T1. Therefore, H2 was not supported. Concerning H3, as illustrated in Table 2, the interaction of supervisor support and organizational identification was significant (estimate = −0.28, SE= 0.10, CI [−0.49, −0.08], *p* = 0.01).

**Figure 1:**
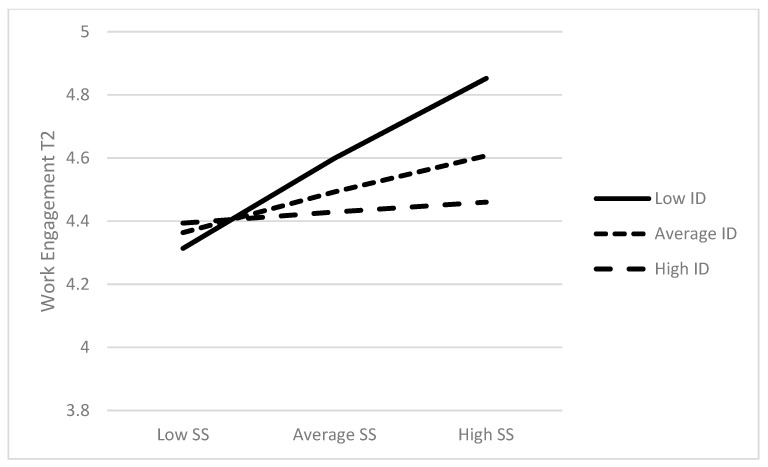
Simple slopes of the direct effect of T1 social support (SS) on T2 work engagement at high, medium, and low levels of T1 organizational identification (ID).

In Figure 1’s simple slope analysis [71], the fact that perceived social support was strongly and positively related to work engagement for employees with lower (estimate = 0.51, SE = 0.16, CI [0.21, 0.82], *p* = 0.001) identification is clearly portrayed, whereas the relationship between social support and work engagement was weaker and not significant for employees with medium (estimate = 0.23, SE= 0.14, CI [−0.04, 0.50], *p* = 0.09) and higher identification (estimate = 0.06, SE= 0.16, CI [−0.26, 0.38], *p* = 0.69). Therefore, H3 was fully supported.

Following the recommendations of Becker [65], the analyses were run with and without the controls in order to exclude the controls as a potential explanation of the results. The pattern of our findings remained substantially the same. Without including demographic variables as covariates, the relationship between social support and work engagement was also significant for medium levels of identification (*p* = 0.04).

## 6. Discussion

Discovering what factors facilitate the development of employees’ engagement is a relevant topic both for researchers and organizations because it improves people’s physical and psychological health [72] but also increases their personal and organizational performance [4,73,74]. For this reason, the present study was developed around some key constructs (i.e., social support and organizational identification) in determining work engagement. Moreover, the present research aimed to study how social support and organizational identification interact to predict work engagement longitudinally. To be specific, we hypothesized (H1) that when employees can rely on their supervisors’ and colleagues’ support, they will be more engaged in their work three months later. Using a longitudinal study design, our hypothesis was confirmed, since we identified a positive association between social support and work engagement. In fact, as expected, when social support increased, work engagement also increased in intensity after controlling for engagement at the baseline (T1). Our results are in line with the reference literature [16,20,75] and with the assumptions of social exchange theory. Precisely, the concept of social reciprocity claims that employees are motivated to perform better and to possess positive attitudes at work (for example, being more engaged) when they feel they have received the correct treatment—something that is not only based on remuneration, career opportunities, and job security. For this reason, the social relationships and the emotional exchanges that come from them take on great importance. Our findings are also coherent with several previous studies developed within the framework of the JD-R model, where social interactions (social support) can be seen as a type of job resource that leads to work engagement [4,12,18,19].

To understand the mechanisms that determine the genesis of work engagement, the second hypothesis of our research relies on the analysis of the association between organizational identification and work engagement. Specifically, according to social identity theory and previous researches [35,39,45,52,54], in H2, we hypothesized that employees who highly identify with their organization are more easily engaged at work. This is because they recognize the organization’s goals as their own and are encouraged to commit themselves to achieving these goals and performing in agreement with the organization’s values and norms [76]. This finding is also supported by Ashforth et al. [33], who underlined that, when a specific membership becomes salient, employees tend to show favoritism for that membership in terms of greater engagement and cooperation.

Although we found a positive correlation between identification and work engagement at both times, we did not find a significant relationship between T1 identification and T2 work engagement when we controlled for work engagement at T1. Therefore, our second hypothesis was not confirmed. Despite numerous studies, corroborated by the meta-analyses conducted by Steffens and colleagues [48], showing that identification with one’s organization tends to increase job satisfaction and well-being, findings from published studies reported inconsistent results too [76,77]. Several motives might underline these conflicting results, such as those of the present research. Firstly, we should consider that most of the previous studies are cross-sectional in nature. As underlined by Maxwell and Cole [5], when utilizing a longitudinal approach, even strong relationships established in cross-sectional evidence may disappear. Another justification might be that employees’ well-being could be a more distal consequence of identification, and this relationship could be mediated by other psychosocial mechanisms. For example, Avanzi and colleagues [76,78] found a nonlinear path between identification and workaholism (work addiction). These authors showed that workaholism nonlinearly mediated the relationship between organizational identification and psychological distress, meaning that identification reduced psychological distress through a reduction in work addiction (i.e., workaholism), but only for low levels of identification. On the contrary, when the levels of organizational identification become higher, this leads to an increase in workaholism and an increase in psychological distress. These findings are consistent with other recent empirical investigations [79], which found that, contrary to their expectations, organizational identification did not act as a predictor of work engagement when simultaneously tested in relation to community experiences, identity, and commitment. Therefore, our research supports the likelihood that this construct may work in more complex ways than has been examined in the literature to date.

Finally, starting from the cross-sectional findings of van Knippenberg et al. [50] and Avanzi et al. [54] concerning withdrawal behaviors, we predicted (H3) that organizational identification would buffer the direct effect of perceived support on work engagement. To this end, a longitudinal analysis was conducted using a positive outcome (i.e., work engagement) instead of negative ones (i.e., absenteeism and turnover intentions). Through a longitudinal design, this study extends the results of Avanzi et al. [54] and van Knippenberg et al. [50] on withdrawal behaviors, since we demonstrated that, for highly identified employees, the bond between support and work engagement is weaker (i.e., not significant) than for less identified employees. In other words, when employees strongly identify with their organizations, their evaluation of the social support received from colleagues and supervisors becomes less critical in determining their work engagement over time. Individuals with high levels of organizational identification may be more likely to find meaning in their work itself, regardless of the level of social support they receive. From the social identity perspective, a strong identification makes employee and the organization two entities less psychological separated, and in this case the role and the quality of social exchange relationships, such as colleagues support, becomes less salient in fostering employee’s engagement. On the contrary, social support becomes more critical for employees who are less identified. In this case, employees perceive the organization as a more distinct entity from themselves (i.e., less identification), and the exchange relationships become more salient. As a consequence, a positive evaluation about the quality of these relationships in work context may represent a crucial job resource able to motivate employees to reciprocate this relation in terms of more effort, dedication, and commitment.

Our findings are consistent with previous research on the role of social support in promoting work engagement [16]. Starting from the social exchange theory, good quality of work relationships (e.g., high social support) should enhance employees’ work engagement. Moreover, providing support and appreciation among colleagues can contribute to employee well-being and job performance. However, this study extends this research by demonstrating that the beneficial effect of social support on work engagement depends on employees’ organizational identification. Higher identified employees are more loyal and more committed with their organization because they internalize organizational values and goals [50], and for this they are less concerned about the quality of the work relationships. On the contrary, less identified employees will greatly benefit from a positive relationship at work. Organizations may advantage from implementing interventions to promote social support among their employees, especially for those who may be less identified with the organization.

### 6.1. Practical Implication

From a practical standpoint, organizations and managers can foster employees’ work engagement by designing interventions focused on increasing social support among their employees. Specifically, this can be carried out through the creation of group interventions designed to stimulate social exchange and joint decision making. This type of intervention has been shown to be particularly effective (see [80] for a meta-analysis) because participation in group activities leads participants to develop a sense of identity with their group. Being part of such groups gives people the opportunity to collaborate with others to achieve a common goal, such as improving a certain component of the work environment or solving a certain work-related problem and allowing them to meet needs related to a sense of belonging, purpose, and meaning. This process can increase job resources, social support, and work engagement [81]. Therefore, organizations should increase team spirit among their employees by implementing group- and organization-wide activities and providing group-oriented rewards and incentives. In addition, they could encourage initiatives designed to increase employees’ feeling of being part of a family in the workplace through, for example, ceremonies and other joint events [82]. These interventions would then go a long way toward both creating positive social relationships and increasing organizational identification. For the development of organizational identification, along with the interventions described above, the role of leadership is relevant. The leader could shape and promote employees’ attitudes and behaviors by being a prototypical member of the same organization [83,84,85]. Organizations could select and develop leaders who perform best in terms of representing organizational values, such as in terms of prototypical identity (e.g., “the leader is a model member of the organization”) using 360-degree feedback systems through which both employees and their employer can identify the right leader for the organization. In this sense, in a cross-cultural study, van Dick and colleagues [85] found evidence that prototypical leadership was able to create the collective sense of “us” within organizations, fostering the organizational members’ engagement and reducing stress. This is because a positive leadership style increases employees’ organizational belongingness.

### 6.2. Limitations

Our research also has some limitations that should be mentioned. First, the longitudinal sample was not particularly large. This condition is quite frequent in longitudinal designs [86] but represents a limitation in terms of the results’ generalizability. Thus, future studies should test this hypothesis with larger samples. Moreover, the significance of our results was controlled by using the bootstrapping method, which is particularly appropriate for small samples. Additionally, the study was entirely based on self-report data. Finally, although we adopted widely used scales, we were forced to drop one item in both the support and identification instruments due to its low reliability. In our view, future studies should analyze the impact of multiple foci of identification, over and above organizational identification, since they may show specific relations with different outcomes, thus giving a more complete picture of how this relates to attitudes and behaviors related to work (e.g., [47]).

## 7. Conclusions

Grounded in the social exchange and social identity theories, and by using a longitudinal design, this study sought to examine the role of social support and organizational identification as essential drivers of work engagement.

In summary, our research confirmed the role of social support in the construction of employees’ work engagement; although, contrary to our expectations, we did not find a direct path from organizational identification measured at T1 to work engagement at T2. However, we demonstrated another mechanism that involves both social support and organizational identification in explaining work engagement over time. Based on the results, we think that this research contributes to the improvement of theoretical and practical implications and offers intriguing directions for both future studies and organizational practice.

## Figures and Tables

**Table 1:** Means, standard deviations, Cronbach alpha values (in parentheses), and correlations among the study variables (N = 150).

Variable	M	SD	1	2	3	4	5	6	7	8
1. Gender (1 = female)	0.68	0.47	(-)							
2. Job tenure	11.91	10.06	−0.00	(-)						
3. Organization 2 (bank)	0.18	0.39	0.05	0.71 **	(-)					
4. Organization 3 (social cooperative)	0.35	0.48	0.44 **	−0.23 **	−0.34 **	(-)				
5. Social support T1	3.28	0.57	0.05	−0.21 *	−0.03	0.18 *	(0.87)			
6. Identification T1	3.64	0.81	0.22 **	−0.09	−0.07	0.28 **	0.32 **	(0.69)		
7. Work engagement T1	4.44	1.30	0.22 **	−0.25 **	−0.22 **	0.27 **	0.50 **	0.47 **	(0.94)	
8. Work engagement T2	4.45	1.25	0.08	−0.20 *	−0.20 *	0.27 **	0.51 **	0.38 **	0.81 **	(0.95)

Note: * *p* < 0.05; ** *p* < 0.01.

**Table 2:** Path coefficients, standard errors, and 95% bias-corrected confidence intervals predicting T2 work engagement (N = 150).

	*Dependent Variable* Work Engagement T2				
*Predictor Variables*	Coefficient	SE	*p*	LLCI	ULCI
Social support (SS) T1	0.27	0.14	0.04	0.0056	0.5423
Organizational identification (ID) T1	−0.09	0.10	0.38	−0.2921	0.1110
SSxID	−0.28	0.10	0.01	−0.4852	−0.0770
*Control variables*					
Gender (1 = female)	−0.22	0.17	0.19	−0.561	0.1144
Job tenure	0.00	0.01	0.95	−0.0183	0.0194
Organization 2 (bank)	−0.14	0.28	0.61	−0.6987	0.4105
Organization 3 (social cooperative)	0.20	0.18	0.26	−0.1483	0.5484
Work engagement T1	0.68	0.07	0.00	0.5368	0.8254

R^2^ = 0.69; F (HC3)_(8; 131)_ = 41.21, *p* < 0.001.

## Data Availability

The data presented in this study are available on request from the corresponding author.

## References

[1] Bakker A.B., Burke R.J., Cooper C.L. (2008). The Peak Performing Organization.

[2] Seligman M.E., Csikszentmihalyi M. (2000). Positive Psychology. An Introduction. Am. Psychol..

[3] Schaufeli W.B., Bakker A.B., Bakker A.B., Leiter M. (2011). Defining and Measuring Work Engagement: Bringing Clarity to the Concept. Work Engagement: A Handbook of Essential Theory and Research.

[4] Mazzetti G., Robledo E., Vignoli M., Topa G., Guglielmi D., Schaufeli W.B. (2021). Work Engagement: A Meta-Analysis Using the Job Demands-Resources Model. Psychol. Rep..

[5] Maxwell S.E., Cole D.A. (2007). Bias in Cross-Sectional Analyses of Longitudinal Mediation. Psychol. Methods.

[6] Bakker A.B., Demerouti E. (2007). The Job Demands-Resources Model: State of the Art. J. Manag. Psychol..

[7] Khoreva V., van Zalk M. (2016). Antecedents of Work Engagement among High Potential Employees. Career Dev. Int..

[8] Monje Amor A., Xanthopoulou D., Calvo N., Abeal Vázquez J.P. (2021). Structural Empowerment, Psychological Empowerment, and Work Engagement: A Cross-Country Study. Eur. Manag. J..

[9] Babcock-Roberson M.E., Strickland O.J. (2010). The Relationship Between Charismatic Leadership, Work Engagement, and Organizational Citizenship Behaviors. J. Psychol..

[10] Simbula S., Guglielmi D., Schaufeli W.B., Depolo M. (2013). An Italian Validation of the Utrecht Work Engagement Scale: Characterization of Engaged Groups in a Sample of Schoolteachers. Boll. Di Psicol. Appl..

[11] Bakker A.B., Demerouti E. (2017). Job Demands–Resources Theory: Taking Stock and Looking Forward. J. Occup. Health Psychol..

[12] Schaufeli W.B., Leiter M.P., Maslach C. (2009). Burnout: 35 Years of Research and Practice. Career Dev. Int..

[13] Schaufeli W.B., Bakker A.B., van Rhenen W. (2009). How Changes in Job Demands and Resources Predict Burnout, Work Engagement, and Sickness Absenteeism. J. Organ. Behav..

[14] Lesener T., Gusy B., Wolter C. (2019). The Job Demands-Resources Model: A Meta-Analytic Review of Longitudinal Studies. Work Stress.

[15] Lesener T., Gusy B., Jochmann A., Wolter C. (2020). The Drivers of Work Engagement: A Meta-Analytic Review of Longitudinal Evidence. Work Stress.

[16] Jolly P.M., Kong D.T., Kim K.Y. (2021). Social Support at Work: An Integrative Review. J. Organ. Behav..

[17] Martín Arribas M.C.C. (2007). Estrés Relacionado Con El Trabajo (Modelo de Demanda-Controlapoyo Social) y Alteraciones En La Salud: Una Revisión de La Evidencia Existente. Enferm. Intensiva.

[18] Simbula S., Guglielmi D., Schaufeli W.B. (2011). A Three-Wave Study of Job Resources, Self-Efficacy, and Work Engagement among Italian Schoolteachers. Eur. J. Work. Organ. Psychol..

[19] Geisler M., Berthelsen H., Muhonen T. (2019). Retaining Social Workers: The Role of Quality of Work and Psychosocial Safety Climate for Work Engagement, Job Satisfaction, and Organizational Commitment. Hum. Serv. Organ. Manag. Leadersh. Gov..

[20] Mascarenhas C., Galvão A.R., Marques C.S. (2022). How Perceived Organizational Support, Identification with Organization and Work Engagement Influence Job Satisfaction: A Gender-Based Perspective. Adm. Sci..

[21] Yoon K.H., Lee C.Y., Peng N.L. (2021). Burnout and Work Engagement among Dispatch Workers in Courier Service Organizations. Asia-Pac. Soc. Sci. Rev..

[22] Lockwood N.R. (2007). Leveraging Employee Engagement for Competitive Advantage: HR’s Strategic Role. HR Mag..

[23] Eisenberger R., Huntington R., Hutchison S., Sowa D. (1986). Perceived Organizational Support. J. Appl. Psychol..

[24] Blau P.M. (2017). Exchange and Power in Social Life.

[25] Emerson R.M. (1976). Social Exchange Theory. Annu. Rev. Sociol.

[26] Homans G.C. (1958). Social Behavior as Exchange. Am. J. Sociol..

[27] Ladebo O.J. (2008). Perceived Supervisory Support and Organisational Citizenship Behaviours: Is Job Satisfaction a Mediator?. S. Afr. J. Psychol..

[28] Liu Z., Min Q., Zhai Q., Smyth R. (2016). Self-Disclosure in Chinese Micro-Blogging: A Social Exchange Theory Perspective. Inf. Manag..

[29] Emerson R.M. (1962). Power-Dependence Relations. Sociol. Rev..

[30] Cook K.S., Cheshire C., Rice E.R.W., Nakagawa S. (2013). Social Exchange Theory. Handbooks of Sociology and Social Research.

[31] Ancarani A., di Mauro C., Giammanco M.D., Giammanco G. (2018). Work Engagement in Public Hospitals: A Social Exchange Approach. Int. Rev. Public Adm..

[32] Tajfel H., Turner J., Austin W.G., Worchel S. (1979). An Integrative Theory of Intergroup Conflict. The Social Psychology of Intergroup Relations.

[33] Ashforth B.E., Harrison S.H., Corley K.G. (2008). Identification in Organizations: An Examination of Four Fundamental Questions. J. Manag..

[34] Ashforth B.E., Mael F. (1989). Social Identity Theory and the Organization. Acad. Manag. Rev..

[35] Avanzi L., Fraccaroli F., Castelli L., Marcionetti J., Crescentini A., Balducci C., van Dick R. (2018). How to Mobilize Social Support against Workload and Burnout: The Role of Organizational Identification. Teach. Teach. Educ..

[36] Eriş A., Kökalan Ö. (2022). The Moderating Effect of Organizational Identification on the Relationship Between Organizational Role Stress and Job Satisfaction. Front. Psychol..

[37] Valencia M.N., Regina M., de Gracia L. (2022). The Moderating Role of Organizational Identification in the Relationship Between Job Demands and Burnout. J. Stress Trauma Anxiety Resil..

[38] van Dick R., Haslam A.S., Jetten J., Haslam C., Haslam S.A. (2012). Stress and Well-Being in the Workplace: Support for Key Propositions from the Social Identity Approach. The Social Cure: Identity, Health and Well-Being.

[39] Ötken A.B., Erben G.S. (2010). Investigating the Relationship Between Organizational Identification and Work Engagement and the Role of Supervisor Support. Gazi Üniversitesi İktisadi Ve İdari Bilim. Fakültesi Derg..

[40] Fallatah F., Laschinger H.K.S., Read E.A. (2017). The Effects of Authentic Leadership, Organizational Identification, and Occupational Coping Self-Efficacy on New Graduate Nurses’ Job Turnover Intentions in Canada. Nurs. Outlook.

[41] van Dick R., Christ O., Stellmacher J., Wagner U., Ahlswede O., Grubba C., Hauptmeier M., Hohfeld C., Moltzen K., Tissington P.A. (2004). Should I Stay or Should I Go? Explaining Turnover Intentions with Organizational Identification and Job Satisfaction*. Br. J. Manag..

[42] O’Keefe D.F., Peach J.M., Messervey D.L. (2019). The Combined Effect of Ethical Leadership, Moral Identity, and Organizational Identification on Workplace Behavior. J. Leadersh. Stud..

[43] Karanika-Murray M., Duncan N., Pontes H.M., Griffiths M.D. (2015). Organizational Identification, Work Engagement, and Job Satisfaction. J. Manag. Psychol..

[44] Piccoli B., Callea A., Urbini F., Chirumbolo A., Ingusci E., de Witte H. (2017). Job Insecurity and Performance: The Mediating Role of Organizational Identification. Pers. Rev..

[45] Avanzi L., Perinelli E., Bressan M., Balducci C., Lombardi L., Fraccaroli F., van Dick R. (2021). The Mediational Effect of Social Support between Organizational Identification and Employees’ Health: A Three-Wave Study on the Social Cure Model. Anxiety Stress Coping.

[46] Lee E.-S.S., Park T.-Y.Y., Koo B. (2015). Identifying Organizational Identification as a Basis for Attitudes and Behaviors: A Meta-Analytic Review. Psychol. Bull..

[47] Riketta M. (2005). Organizational Identification: A Meta-Analysis. J. Vocat. Behav..

[48] Steffens N.K., Haslam S.A., Schuh S.C., Jetten J., van Dick R. (2017). A Meta-Analytic Review of Social Identification and Health in Organizational Contexts. Personal. Soc. Psychol. Rev..

[49] van Dick R., Grojean M.W., Christ O., Wieseke J. (2006). Identity and the Extra Mile: Relationships between Organizational Identification and Organizational Citizenship Behaviour. Br. J. Manag..

[50] van Knippenberg D., van Dick R., Tavares S. (2007). Social Identity and Social Exchange: Identification, Support, and Withdrawal From the Job. J. Appl. Soc. Psychol..

[51] Kesen M. (2016). Linking Organizational Identification with Individual Creativity: Organizational Citizenship Behavior as a Mediator. J. Yaşar Univ..

[52] He H., Brown A.D. (2013). Organizational Identity and Organizational Identification: A Review of the Literature and Suggestions for Future Research. Group Organ. Manag..

[53] Kanjanakan P., Zhu D., Doan T., Kim P.B. (2021). Taking Stock: A Meta-Analysis of Work Engagement in the Hospitality and Tourism Context. J. Hosp. Tour. Res..

[54] Avanzi L., Fraccaroli F., Sarchielli G., Ullrich J., van Dick R. (2014). Staying or Leaving: A Combined Social Identity and Social Exchange Approach to Predicting Employee Turnover Intentions. Int. J. Product. Perform. Management.

[55] Atkinson L., Niccols A., Paglia A., Coolbear J., Parker K.C.H., Poulton L., Guger S., Sitarenios G. (2016). A Meta-Analysis of Time between Maternal Sensitivity and Attachment Assessments: Implications for Internal Working Models in Infancy/Toddlerhood. J. Soc. Pers. Relationsh..

[56] Riketta M. (2008). The Causal Relation Between Job Attitudes and Performance: A Meta-Analysis of Panel Studies. J. Appl. Psychol..

[57] Mael F., Ashforth B.E. (1992). Alumni and Their Alma Mater: A Partial Test of the Reformulated Model of Organizational Identification. J. Organ. Behav..

[58] Bergami M., Bagozzi R.P. (2000). Self-Categorization, Affective Commitment and Group Self-Esteem as Distinct Aspects of Social Identity in the Organization. Br. J. Soc. Psychol..

[59] Schaufeli W.B., Bakker A.B., Salanova M. (2006). The Measurement of Work Engagement With a Short Questionnaire. Educ. Psychol. Meas..

[60] Edwards J.A., Webster S., van Laar D., Easton S. (2008). Psychometric Analysis of the UK Health and Safety Executive’s Management Standards Work-Related Stress Indicator Tool. Work Stress.

[61] Toderi S., Balducci C., Edwards J.A., Sarchielli G., Broccoli M., Mancini G. (2013). Psychometric Properties of the UK and Italian Versions of the HSE Stress Indicator Tool. Eur. J. Psychol. Assess..

[62] Field A. (2005). Discovering Statistics Using SPSS.

[63] Ng T.W.H., Feldman D.C. (2010). The Relationships of Age with Job Attitudes: A Meta-Analysis. Pers. Psychol..

[64] Hakanen J.J., Ropponen A., Schaufeli W.B., de Witte H. (2019). Who Is Engaged at Work?. J. Occup. Environ. Med..

[65] Becker T.E. (2005). Potential Problems in the Statistical Control of Variables in Organizational Research: A Qualitative Analysis With Recommendations. Organ. Res. Methods.

[66] Hayes F.A. (2013). Introduction to Mediation, Moderation, and Conditional Process Analysis: A Regression-Based Approach.

[67] Hayes A.F., Cai L. (2007). Using Heteroskedasticity-Consistent Standard Error Estimators in OLS Regression: An Introduction and Software Implementation. Behav. Res. Methods.

[68] Long J.S., Ervin L.H. (2000). Using Heteroscedasticity Consistent Standard Errors in the Linear Regression Model. Am. Stat..

[69] Robinson C.D., Tomek S., Schumacker R.E. (2013). Tests of Moderation Effects: Difference in Simple Slopes versus the Interaction Term. Mult. Linear Regres. Viewp..

[70] Frazier P.A., Tix A.P., Barron K.E. (2004). Testing Moderator and Mediator Effects in Counseling Psychology Research. J. Couns. Psychol..

[71] Toothaker L.E., Aiken L.S., West S.G. (1994). Multiple Regression: Testing and Interpreting Interactions. J. Oper. Res. Soc..

[72] Roelen C.A.M., van Hoffen M.F.A., Groothoff J.W., de Bruin J., Schaufeli W.B., van Rhenen W. (2015). Can the Maslach Burnout Inventory and Utrecht Work Engagement Scale Be Used to Screen for Risk of Long-Term Sickness Absence?. Int. Arch. Occup. Environ. Health.

[73] Alessandri G., Borgogni L., Schaufeli W.B., Caprara G.V., Consiglio C. (2015). From Positive Orientation to Job Performance: The Role of Work Engagement and Self-Efficacy Beliefs. J. Happiness Stud..

[74] Bakker A.B., Albrecht S. (2018). Work Engagement: Current Trends. Career Dev. Int..

[75] Cao X., Chen L. (2019). Relationships among Social Support, Empathy, Resilience and Work Engagement in Haemodialysis Nurses. Int. Nurs. Rev..

[76] Avanzi L., van Dick R., Fraccaroli F., Sarchielli G. (2012). The Downside of Organizational Identification: Relations between Identification, Workaholism and Well-Being. Work Stress.

[77] Bizumic B., Reynolds K.J., Turner J.C., Bromhead D., Subasic E. (2009). The Role of the Group in Individual Functioning: School Identification and the Psychological Well-Being of Staff and Students. Appl. Psychol..

[78] Avanzi L., Savadori L., Fraccaroli F., Ciampa V., van Dick R. (2022). Too-Much-of-a-Good-Thing? The Curvilinear Relation between Identification, Overcommitment, and Employee Well-Being. Curr. Psychol..

[79] Boyd N.M., Nowell B. (2020). Sense of Community, Sense of Community Responsibility, Organizational Commitment and Identification, and Public Service Motivation: A Simultaneous Test of Affective States on Employee Well-Being and Engagement in a Public Service Work Context. Public Manag. Rev..

[80] Knight C., Patterson M., Dawson J. (2017). Building Work Engagement: A Systematic Review and Meta-Analysis Investigating the Effectiveness of Work Engagement Interventions. J. Organ. Behav..

[81] Nielsen K. (2013). Review Article: How Can We Make Organizational Interventions Work? Employees and Line Managers as Actively Crafting Interventions. Hum. Relat..

[82] Steffens N.K., LaRue C.J., Haslam C., Walter Z.C., Cruwys T., Munt K.A., Haslam S.A., Jetten J., Tarrant M. (2019). Social Identification-Building Interventions to Improve Health: A Systematic Review and Meta-Analysis. Health Psychol. Rev..

[83] Bracht E.M., Monzani L., Boer D., Haslam S.A., Kerschreiter R., Lemoine J.E., Steffens N.K., Akfirat S.A., Avanzi L., Barghi B. (2022). Innovation across Cultures: Connecting Leadership, Identification, and Creative Behavior in Organizations. Appl. Psychol..

[84] van Dick R., Hirst G., Grojean M.W., Wieseke J. (2007). Relationships between Leader and Follower Organizational Identification and Implications for Follower Attitudes and Behaviour. J. Occup. Organ. Psychol..

[85] van Dick R., Cordes B.L., Lemoine J.E., Steffens N.K., Haslam S.A., Akfirat S.A., Ballada C.J.A., Bazarov T., Aruta J.J.B.R., Avanzi L. (2021). Identity Leadership, Employee Burnout and the Mediating Role of Team Identification: Evidence from the Global Identity Leadership Development Project. Int. J. Environ. Res. Public Health.

[86] Ford M.T., Tetrick L.E. (2011). Relations among Occupational Hazards, Attitudes, and Safety Performance. J. Occup. Health Psychol..

